# Plasma biomarkers, brain amyloid pathology, and cortical thickness in a racially diverse community cohort: the Human Connectome Project

**DOI:** 10.1002/alz70856_107562

**Published:** 2026-01-09

**Authors:** Shayna Brodman

**Affiliations:** ^1^ University of Pittsburgh Medical Center, Pittsburgh, PA, USA

## Abstract

**Background:**

Plasma biomarkers are critical for early detection and treatment of Alzheimer's disease (AD). We assessed plasma biomarker association and classification capacity against neuroimaging measures.

**Method:**

In the racially diverse Human Connectome Project (HCP; *n* = 218, 15% Aβ‐PET‐positive) Aβ‐PET and cortical thickness (neurodegeneration) were related to plasma biomarkers (*p*‐tau181, *p*‐tau217 [Janssen and ALZpath], *p*‐tau231, GFAP, NfL, Aβ42/Aβ40).

**Result:**

Plasma *p*‐tau217 accurately identified abnormal Aβ‐PET (: AUC=0.9145, 95% CI=[0.8367, 0.9923]) followed by GFAP and Aβ42/40 ratio (GFAP (AUC=0.8529, 95% CI = [0.7485, 0.9573], Aβ42/40 (AUC = 0.7962, 95% CI = [0.6581, 0.9346]). All biomarkers performed poorly to identify cortical thickness, but were increased according to combined Aβ‐PET‐neurodegeneration profiles. Correlations of *p*‐tau217, *p*‐tau181, and Aβ42/40 with Aβ‐PET were stronger in self‐identified non‐Hispanic Whites vs. Black/African Americans.

**Conclusion:**

Plasma biomarkers identify brain Aβ pathology in the community. However, consideration of potential racial/ethnic differences in brain‐to‐blood biomarker correlations should be critically examined and addressed to enable widespread cross‐population applications.

